# INTRAUTERINE GROWTH AND THE VITAMIN E STATUS OF FULL-TERM AND PRETERM NEWBORNS

**DOI:** 10.1590/1984-0462/;2019;37;3;00003

**Published:** 2019-05-09

**Authors:** Alyne Batista da Silva, Jeane Franco Pires Medeiros, Mayara Santa Rosa Lima, Amanda Michelly Braga da Mata, Eva Débora de Oliveira Andrade, Danielle Soares Bezerra, Mônica Maria Osório, Roberto Dimenstein, Karla Danielly da Silva Ribeiro

**Affiliations:** a Universidade Federal do Rio Grande do Norte, Natal, RN, Brazil.; b Universidade Federal do Rio Grande do Norte, Santa Cruz, RN, Brazil.; c Universidade Federal de Pernambuco, Recife, PE, Brazil.

**Keywords:** Alpha-Tocopherol;Premature infants, Nutritional status;Umbilical cord, Alfa-Tocoferol, Recém-nascido prematuro, Estado nutricional, Cordão umbilical

## Abstract

**Objective:**

To determine the concentration of alpha-tocopherol in umbilical cord serum of full-term and preterm newborns, in order to assess the nutritional status of both groups in relation to the vitamin and its possible correlation with intrauterine growth.

**Methods:**

A cross-sectional observational study conducted with 140 newborns, of which 64 were preterm and 76 were full-term. They did not have any malformations, they came from healthy mothers, who were nonsmokers, and delivered a single baby. Intrauterine growth was evaluated by weight-to-gestational age at birth, using Intergrowth-21^st^. Thealpha-tocopherol levels of umbilical cord serum were analyzed by High Performance Liquid Chromatography.

**Results:**

The mean concentration of alpha-tocopherol in umbilical cord serum for preterm and full-term infants was 263.3±129.5 and 247.0±147.6 µg/dL (p=0.494). In the preterm group, 23% were small for gestational age, whereas in the full-term group, this percentage was only 7% (p=0.017). Low levels of vitamin E were found in 95.3% of preterm infants and 92.1% of full-term infants. There was no correlation between alpha-tocopherol levels and weight to gestational age Z score (p=0.951).

**Conclusions:**

No association was found between alpha-tocopherol levels and weight to gestational age at birth. Intrauterine growth restriction was more frequent in preterm infants and most infants had low levels of vitamin E at the time of delivery.

## RESUMO

**Objetivo:**

Determinar a concentração de alfatocoferol em soro de cordão umbilical de recém-nascidos a termo e pré-termo, a fim de avaliar o estado nutricional de ambos os grupos com relação a essa vitamina e sua possível correlação sobre o crescimento intrauterino.

**Métodos:**

Estudo observacional de caráter transversal realizado com 140 recém-nascidos, 64 pré-termo e 76 a termo, sem malformações, oriundos de mães saudáveis, não fumantes e com parto de concepto único. O crescimento intrauterino foi avaliado pelo índice peso por idade gestacional ao nascer, utilizando a Intergrowth-21^st^. Os níveis de alfatocoferol do soro do cordão umbilical foram analisados por cromatografia líquida de alta eficiência.

**Resultados:**

A concentração média de alfatocoferol no soro do cordão umbilical para recém-nascidos pré-termo e a termo foi de, respectivamente, 263,3±129,5 e 247,0±147,6 µg/dL (p=0,494). Baixos níveis de vitamina E foram encontrados em 95,3% dos prematuros e em 92,1% dos neonatos a termo. Nogrupo pré-termo, 23% eram pequenos para a idade gestacional, enquanto no grupo a termo esse percentual foi de apenas 7% (p=0,017). Não houve correlação entre os níveis de alfatocoferol e o escore Z de peso para idade gestacional (p=0,951).

**Conclusões:**

Não foi encontrada associação entre os níveis de alfatocoferol e a adequação do peso à idade gestacional ao nascer. A restrição do crescimento intrauterino foi mais frequente nos nascidos pré-termo, e a maioria dos recém-nascidos apresentou níveis baixos de vitamina E no momento do parto.

## INTRODUCTION

Newborns are considered an at-risk group for vitamin E deficiency, considering that the transplacental transfer of alpha-tocopherol is limited. This can result in low serum and tissue levels of Vitamin E at birth, especially in premature newborns.^1^ ^,^ ^2^ ^,^ ^3^ ^,^ ^4^

Low serum levels of alpha-tocopherol are associated with the development of edemas, thrombocytosis, and hemolytic anemia, which can result in spinocerebellar degeneration.^5^ They may also result in cardiomyopathy as a consequence of probable muscular degeneration.^6^ Another possible consequence of this vitamin deficiency is its restriction on the intrauterine growth of fetuses. This hypothesis is based on the fact that vitamin E has the ability to increase the release of prostaglandins I_2_ and E_2_,^7^ which are vasodilators compounds, and could possibly help to improve the blood supply to the fetus. Considering this, low levels of alpha-tocopherol could consequently compromise the supply of nutrients to the fetus, interfering in its growth.^8^

Intrauterine growth restriction (IUGR) is one of the main causes of neonatal morbidity and mortality, and has possible repercussions in adulthood, especially with regard to cardiovascular diseases.^9^ IUGR is more prevalent in developing countries, occurring in 7 to 15% of pregnancies. In Brazil, it is estimated that this percentage is between 10 and 15%.^10^ Inaddition, it is reported that preterm infants are five times more likely to present IUGR than those born at term.^9^ In 2015, the Global Health Network released Intergrowth-21^st^, which is currently the most suitable tool for assessing intrauterine growth, by providing up-to-date growth curves for preterm and full-term newborns.^11^ ^,^ ^12^

However, despite the possible role of alpha-tocopherol in aiding the intrauterine development of fetuses, there are still few studies that evaluate this relationship, and consider weight and gestational age at birth. There are reports that full-term newborns with appropriate weight for gestational age (AGA) present higher levels of alpha-tocopherol than infants that are small or large for their gestational age.^10^ ^,^ ^13^ With regard to the preterm infants, this difference was not identified.^13^ Whenconsidering only birth weight, it is observed that the higher the weight, the higher the levels of alpha-tocopherol in the umbilical cord.^4^ ^,^ ^14^ ^,^ ^15^

Thus, considering that premature newborns represent an at-risk group for low alpha-tocopherol serum levels and IUGR at birth, this study aimed to determine the concentration of alpha-tocopherol in the umbilical cord serum of full-term and preterm newborns in order to evaluate the nutritional status of both groups with regard to this vitamin and its possible correlation with intrauterine development.

## METHOD

The study was comprised of 140 newborns, including 64 preterm infants (< 37 weeks) and 76 full-term infants (≥ 37 weeks), who were cared for at two public maternity wards in Rio Grande do Norte: Ana Bezerra University Hospital, located in the city of Santa Cruz, and Januário Cicco Maternity School, located in Natal, from 2013 to 2015. The inclusion criteria were healthy mothers (without a clinical diagnosis of any diseases), who were non-smokers, and delivered one baby without malformations.

The study was observational with a cross-sectional character for convenience. Birth weight, length at birth, and gestational age at birth were consulted in the patients’ medical records. To complement the research and characterize the population, data on maternal age, family income, type of delivery and parity were collected using forms.

The nursing staff at the maternity wards collected 5 mL of umbilical cord blood at the time of the delivery, in dry polyethylene plastic tubes wrapped in laminated paper (to protect against luminosity), and transported in refrigerated containers until they reached the lab. In the laboratory, the blood was centrifuged for 10 minutes (500 xg) to separate the serum, which was cooled until the levels of alpha-tocopherol were determined.

To extract the alpha-tocopherol serum, we used the adapted method proposed by Ortega etal*.* ^16^ For the serum rate, ethyl alcohol 95% was added in the proportion of 1:1. It was then shaken for 1 minute to allow for the proteins to precipitate. Subsequently, 2 mL of hexane were added to extract the lipid fraction. Then, it was stirred for another 1 minute and centrifuged for 10 minutes (500 xg). The Supernatant (~2 mL) was transferred to a new tube, and the operation was repeated two more times until it resulted in ~6 mL of extract. The total extract was evaporated in a water bath at 37°C and was re-diluted in absolute ethanol in order to apply 20 µL of it in high performance liquid chromatography (HPLC).

The mobile phase used in HPLC was 100% methanol with a 1 mL/min flow. Alpha-tocopherol level was monitored at a wavelength of 292 nm. The analysis took place in the LC-20 chromatograph at Shimadzu, and was coupled to the SPD-20A Shimadzu UV-VIS detector and the C18 LiChrospher^®^ 100 Column RP-18 (5 µm) (Merck, Darmstadt, Germany). For data processing, we used the LC solution^®^ software (Shimadzu Corporation, Kyoto, Japan).

The alpha-tocopherol was identified and quantified in the samples by comparing the retention time and the peak area obtained by the previous application of the tocopherol level standard. The concentration of the standard was confirmed by the specific extinction coefficient for alpha-tocopherol (ε1%, 1 cm=75.8 to 292 nm) in absolute ethanol.^17^ Thedata were expressed in punctual and relative frequencies and alpha-tocopherol in ­µg/­dL with mean and standard deviation. Alpha-tocopherol levels below 500 µG/DL were considered to be low.^18^

Intrauterine growth was evaluated using the anthropometric indices of birth weight and length at birth by gestational age, using the new growth curves of intergrowth-21^st^.^19^ Dataon birth weight, birth length and gestational age were inserted into the Intergrowth-21^st^ software (http://intergrowth21.ndog.ox.ac.uk/en/ManualEntry) to calculate the percentile and Z score. Newborns were classified as small for their gestational age (SGA) when the percentile was <10, AGA when the percentile was 10 to 90, and large for gestational age (GIG) when the percentile was >90.^11^ ^,^ ^12^

The continuous variables were verified to be normal using the Kolmogorov-Smirnov test. Pearson’s correlation was used to verify the correlation between the levels of alpha-tocopherol and the Z score of weight for gestational age, since the data presented normal distribution. The chi-square test was used to ascertain the differences in the categorical variables between the preterm and full-term groups, and the Student’s t-test was used to evaluate the average differences in alpha-tocopherol and maternal age between the groups. The data were analyzed in Statistical Package for the Social Sciences(SPSS), version 7.0 (IBM, São Paulo, Brazil). All differences were considered significant when p<0.05.

The study was approved by the Research Ethics Committee of the Federal University of Rio Grande do Norte (CAAE 07416912.8.0000.5537), and all of the new mothers voluntarily signed the free and informed consent form before starting the collections.

## RESULTS

A total of 140 newborns participated in the research - 64 were preterm and 76 were full-term. Weight and length at birth were different between groups, and intrauterine growth restriction was observed in 23% (n=15) of preterm births and in 7% (n=5) of full-term births (p=0.017) (Table 1).

**Table 1:** Characteristics of preterm and full-term newborns included in the study.

	Preterm, n (%)	Full-term, n (%)	p-value
Gestational age (weeks)	33.9±2.5	39.5±1.4	<0.001^a^
Birth weight (g)	2088±624	3281±412	<0.001^a^
Birth length (cm)	43.8±4.5	48.9±1.8	<0.001^a^
Male sex, n (%)	37 (58)	34 (45)	0.123^b^
Weight/GA (Zscore)	-0.38±1.15	0.06±0.94	0.013^a^
Length/GA (Zscore)	-0.53±1.70	-0.20±1.19	0.199^a^
Intrauterine growth			
SGA, n (%)	15 (23)	5 (7)	0.017^b^
AGA, n (%)	45 (70)	64 (84)	
LGA, n (%)	4 (6)	7 (9)	

^a^Student’s t-test; ^bc^hi-square test; GA: gestational age; SGA: small for gestational age; AGA: appropriate for gestational age; LGA: large for gestational age.

**Figure 1:**
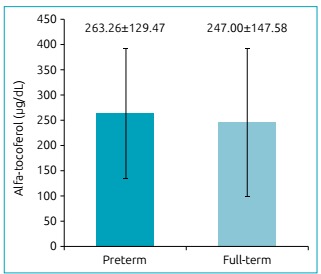
Concentration of alpha-tocopherol in the umbilical cord of preterm and full-term newborns included in the study (p=0.493, Student’s t-test).

There was no significant difference in the levels of alpha-tocopherol in the umbilical cord between the preterm and full-term groups (p=0.493) (Figure 1). The majority of preterm births (95.3%; n=61) and full-term births (92.1%; n=70) demonstrated a low vitamin E status (<500 µg/dL).

There was no correlation between the levels of alpha-tocopherol and the Z-score of the weight to gestational age at birth (r*=*0.005; p=0.951). Considering the SGA, AGA and LGA groups, the average levels of alpha-tocopherol found were, respectively, 243.4, 258.0 and 239.1 µg/dL.

The characterization of the population showed that almost half of the premature newborns (44%) (n=28) had low income (<0.5 minimum wage per capita), while in the full-term group, this result was found in only 8% (n=6; p<0.001) (Table 2). In the full-term group, there were more cases of recent mothers who had had more than one child (55%; n=42; p=0l.031) and who had a normal delivery (87%; n=66; p<0.001) than in the preterm group (Table 2).

**Table 2:** General characteristics of new mothers included in the study.

	Preterm, n (%)	Full-term, n (%)	p-value
Mother’s age (years)	25±7	24±5	0.330^b^
Family income^a^			
Low income, n (%)	28 (44)	6 (8)	<0.001^c^
Satisfactory, n (%)	36 (56)	70 (92)	
Birth type			
Vaginal, n (%)	35 (55)	66 (87)	<0.001^c^
Cesarean, n (%)	27 (42)	10 (13)	
No information, n (%)	2 (3)	0(0)	
Number of previous births			
First birth, n (%)	37 (58)	32 (42)	0.031^c^
Previous births, n (%)	22 (34)	42 (55)	
No information, n (%)	5 (8)	2 (3)	

^a^Low income when family income < 0.5 minimum wage per capita; ^b^Student’s t-test; ^c^chi-square test.

## DISCUSSION

Preterm newborns had a higher frequency of SGA babies, compared to those born at term. The findings were similar to those found in the literature, in which most SGA newborns are premature.^20^ According to the Institute of Medicine in the United States, the greatest weight gain during pregnancy occurs is in the second and third trimester. During this period, pregnant women gain, on average, 420g per week and their fetuses acquire about 80% of their total weight.^21^

Thus, when the child is born prematurely, it does not gain part of the weight it would have during the third trimester, which makes it smaller in relation to full-term newborns.^21^ However, what is worrisome about this situation is not only the fact that premature infants are born smaller, but they are more vulnerable to being born underweight and to the risks of this condition.

A study conducted in Nepal with more than 25,000 women concluded that there is a risk of death that is 12 times higher for premature newborns. When considered premature SGA, the risk increased to 16 times.^22^ In addition to the risk of death, newborns with IUGR may present, in the short term: chronic lung disease, a low Apgar score, need for respiratory support, need for neonatal intensive care, brain injury with long-term consequences, and retinopathy from prematurity.^23^

SGA newborns may also present lower concentrations of alpha-tocopherol in the umbilical cord serum. A study developed in Algeria identified that full-term AGA newborns presented concentrations of alpha-tocopherol in the umbilical cord (528.5µg/dL) that were larger than the SGA newborns (201.7 µg/dL).^10^ Another investigation showed similar results: the concentrations of alpha-tocopherol serum in SGA and LGA newborns were lower than those found in AGA newborns.^13^ Other authors have identified that the higher the birth weight, the higher the concentrations of alpha-tocopherol in the umbilical cord, regardless of the gestational age at birth,^3^ ^,^ ^15^ demonstrating a possible relationship between growth and vitamin E level.

However, in a study that observed full-term infants separately from preterm infants, there were statistical differences in the level of alpha-tocopherol between the AGA and SGA full-term newborns, but not between the SGA and LGA preterm newborns,^14^ most likely because being premature already makes them a vulnerable group at birth with low serum levels of alfa-tocopherol.^1^ ^,^ ^2^ ^,^ ^3^ ^,^ ^4^

However, in the present study, the serum levels of alpha-tocopherol in the umbilical cord were similar among newborns. In relation to preterm births, the values are in agreement with those found in the literature (between 224.8 and 330.0­µg/­dL),^2^ ^,^ ^3^ ^,^ ^4^ ^,^ ^24^ while the concentration of alfa-tocopherol in the full-term newborns was similar to that found in some studies^24^ ^,^ ^25^ and divergent in others,^3^ ^,^ ^4^ perhaps because the different studies use populations of different nationalities. The studies carried out with populations from Egypt and India presented serum alpha-tocopherol averages that were higher than the cutoff point indicated as satisfactory,^3^ ^,^ ^4^ ^,^ ^18^ however, the low sample size, which was a limitation in the present study, may also have contributed to conceal possible differences in the alpha-tocopherol serum between the groups. Additionally, the present study was developed using only one population: residents from the state of Rio Grande do Norte, Brazil.

Among the newborns, only nine (6.4%) presented satisfactory levels of the vitamin. Of these, three (2.1%) were premature. Most of the authors report high percentages of newborns with low levels of alpha-tocopherol at birth. A study that adopted 500 µg/dL as a cutoff point found low levels of the vitamin in 77.4% of preterm infants.^26^ Another study conducted in Tunisia showed that 55.5% of the full-term newborns and 71.3% of the preterm infants were below the cutoff point of alpha-tocopherol serum, 301.7 µg/dL.^27^ It is interesting to note that, although premature infants are at risk for nutritional deficiencies, the levels of alpha-tocopherol were not different from those born at term, a result that disagrees with others reported by authors who have identified concentrations of alpha-tocopherol in preterm infants, when compared to those born at term.^28^

Low serum levels of alpha-tocopherol in newborns are worrisome, since they have become associated with the development of edemas, thrombocytosis, hemolytic anemia, and muscular degeneration, which compromises the nervous system and the myocardium.^5^ ^,^ ^6^ Thus, this condition highlights the importance of monitoring the serum levels of vitamin E, in addition to combating vitamin nutritional deficiency, especially during childhood. The World Health Organization (WHO) considers that the maternal infant supplement (vitamin A, iron and folic acid) programs currently implemented in Brazil have a good cost-benefit, because they are relatively low-cost interventions.^29^ ^,^ ^30^ Thus, conducting interventions with the objective of preventing the conditions associated with vitamin E deficiency could also decrease public health expenditures, since prevention costs are less than the costs of treating diseases.

Despite the alleged role of alpha-tocopherol in improving fetal development through the increase in blood supply and, consequently, of nutrients for the fetus during pregnancy,^7^ ^,^ ^8^ nowadays there is no consensus with regard to the relationship between the level of alpha-tocopherol in the umbilical cord and intrauterine growth. In this study, no association was found between the levels of alpha-tocopherol in umbilical cord blood and intrauterine growth, however the low levels of alpha-tocopherol found in both groups may have limited the results.

It is worth noting that in this study, family income, parity, and type of delivery showed statistical differences between the groups. Only 8% (n=6) of the families of the full-term children were low income, against 44% (n=28) of the families in the preterm group. Low family income can lead to decreased access to medications and medical care, unsatisfactory housing conditions, stressful family contexts, among other factors that may contribute to premature birth.^31^ There is also a higher proportion of women giving birth for the first time with a cesarean section in the preterm group (Table 2). Similar findings were seen in the literature, in which women giving birth for the first time had higher chances of having babies prematurely and having babies with a low birth weight.^31^ Cesarean deliveries are more common in premature births due to the higher risk of mortality and the clinical conditions that usually have indications for this type of surgery, as in the case of extreme ages.^32^

Thus, because of the implications of growth restriction and vitamin E deficiency in newborns, it is essential to study the factors that may be leading to these conditions and the evolution of the nutritional status of these children in the postpartum period, especially when they come from pregnant women with a more vulnerable clinical and socioeconomic profile. Lowlevels of alpha-tocopherol serum, if persistent, can lead to vitamin E deficiency, bringing serious repercussions to the child’s health, including changes in long-term cognitive development.^33^ Theseresults serve as a warning to encourage the monitoring of vitamin E nutritional status following lactation.

It was concluded that preterm infants had higher proportions of IUGR and that, regardless of gestational age, more than 92% of the subjects had low levels of vitamin E at birth. Nodifferences were found between full-term and preterm newborns, nor was there a correlation between intrauterine growth and alpha-tocopherol levels.
